# Non-alcoholic fatty liver disease fibrosis score is a useful index for predicting all-cause mortality in patients with antineutrophil cytoplasmic antibody-associated vasculitis

**DOI:** 10.3389/fmed.2023.1217937

**Published:** 2023-08-29

**Authors:** Jeong Yeop Whang, Pil Gyu Park, Yong-Beom Park, Ji Hye Huh, Sang-Won Lee

**Affiliations:** ^1^Department of Medicine, Yonsei University College of Medicine, Seoul, Republic of Korea; ^2^Division of Rheumatology, Department of Internal Medicine, National Health Insurance Service Ilsan Hospital, Goyang, Republic of Korea; ^3^Division of Rheumatology, Department of Internal Medicine, Yonsei University College of Medicine, Seoul, Republic of Korea; ^4^Institute for Immunology and Immunological Diseases, Yonsei University College of Medicine, Seoul, Republic of Korea; ^5^Division of Endocrinology and Metabolism, Department of Internal Medicine, Hallym University Sacred Heart Hospital, Anyang, Republic of Korea

**Keywords:** non-alcoholic fatty liver disease, fibrosis, score, mortality, antineutrophil cytoplasmic antibody-associated vasculitis

## Abstract

**Background:**

This study investigated whether the non-alcoholic fatty liver disease fibrosis score (NFS) could predict all-cause mortality during follow-up among patients with antineutrophil cytoplasmic antibody-associated vasculitis (AAV).

**Methods:**

The medical records of 256 AAV patients were retrospectively reviewed. AAV patients with clinically critical chronic liver diseases were excluded. NFS was calculated using the following equation: NFS = −1.675 + 0.037 - age + 0.094 – body mass index +1.13 × impaired fasting glucose/diabetes mellitus +0.99 × aspartate aminotransferase/alanine aminotransferase ratio - 0.013 × platelet count - 0.66 × serum albumin.

**Results:**

The median age was 59.0 years, and 35.2% of the patients were male. The median Birmingham Vasculitis Activity Score (BVAS), five-factor score (FFS), and NFS were 12.0, 1.0, and − 4.7, respectively. Of the 256 patients, 33 (12.9%) died. Using the receiver operating characteristic curve, the optimal cut-off of NFS for all-cause mortality was obtained as-3.97. AAV patients with NFS at diagnosis ≥ − 3.97 exhibited a lower cumulative patients’ survival rate than those with NFS at diagnosis <−3.97. The multivariable Cox analysis revealed that NFS at diagnosis ≥ − 3.97 (HR 2.232, 95% CI 1.011, 4.925) was independently associated with all-cause mortality in AAV patients.

**Conclusion:**

This study was the first to demonstrate that NFS at AAV diagnosis was clinically useful in predicting all-cause mortality during follow-up, regardless of both the degree of liver fibrosis and abnormal or normal liver function results.

## 1. Introduction

Non-alcoholic fatty liver disease (NAFLD) is a common cause of chronic liver diseases, with a prevalence of as high as 30% among the general population in developed countries (1). NAFLD has a wide spectrum, from simple liver steatosis to non-alcoholic steatohepatitis (NASH) which may progress to significant liver fibrosis (2). NAFLD has also been reported associated with the occurrence of extrahepatic manifestations including diabetes mellitus (DM), chronic kidney disease, and cardiovascular disease occasionally (3–5). Even in the general population, liver fibrosis was reported to be an independent predictor of mortality (6), which implies that the initial value of NAFLD could be a risk factor for poor outcomes in patients with chronic inflammatory diseases in addition to those with chronic liver diseases.

For confirming the presence or degree of liver fibrosis, a liver biopsy is the standard diagnostic modality; however, not all individuals are suitable candidates to undergo this invasive procedure. For this reason, there have been various scoring systems assessing liver fibrosis introduced, and the NAFLD fibrosis score (NFS) is one of them. NFS comprises six parameters such as age, body mass index (BMI), glucose metabolic abnormality, aspartate aminotransferase (AST)/alanine aminotransferase (ALT) ratio, platelet count, and serum albumin level, is the most well-validated index for liver fibrosis (7). NFS is divided into three ranges based on two cut-off values: −1.455 and 0.675. Negative predictive value for significantly advanced liver fibrosis (F3 ~ F4) was 93% when NFS was < −1.455 (sensitivity 82% and specificity 77%), whereas positive predictive value was up to 90% when NFS is >0.675 (sensitivity 51% and specificity 98%). An indeterminate fibrosis score is defined when NFS is between-1.455 and 0.675 (7, 8).

Antineutrophil cytoplasmic antibody (ANCA)-associated vasculitis (AAV) is a small-vessel vasculitis characterised by necrotising vasculitis with little to no immune deposits and is commonly associated with the presence of ANCA (9, 10). Although AAV has the potential to invade virtually all organs, because it can affect capillaries and adjacent arterioles and venules, it has been rarely reported that AAV can also induce significant liver damage, leading to clinically critical liver fibrosis (11–13). Apart from determining the current status of advanced liver fibrosis, several studies have described various indices for liver fibrosis could predict poor prognosis of AAV during follow-up (14–17). Therefore, it is reasonable to assume that NFS at AAV diagnosis may be associated with all-cause mortality during follow-up among AAV patients. However, to our knowledge, no studies have investigated the clinical utility of NFS in predicting the poor prognosis of AAV. As such, this single-centre study investigated whether NFS at AAV diagnosis could predict all-cause mortality during follow-up among a cohort of AAV patients.

## 2. Methods

### 2.1. Patients

Information from the medical records of 256 patients with AAV, who were from the Severance Hospital ANCA-associated VasculitidEs (SHAVE) cohort, and their available clinical and laboratory data for the equation for NFS at AAV diagnosis were retrospectively reviewed. Inclusion criteria for this cohort are described in the authors’ previous studies (18, 19). Exclusion criteria of this study were as follows: (i) patients who had been diagnosed with clinically critical chronic liver diseases such as B or C viral hepatitis, alcoholic hepatitis, autoimmune hepatitis, and radiologically confirmed liver cirrhosis (8, 11, 20); (ii) patients who had concomitant serious medical conditions including malignancies, infectious diseases requiring hospitalisation, and other vasculitides mimicking AAV; (iii) patients who had not been followed up more than at least 3 months; and (iv) patients who had ever received glucocorticoids or immunosuppressive drugs within 4 weeks prior to AAV diagnosis. The present study was approved by the Institutional Review Board (IRB) of Severance Hospital (Seoul, Republic of Korea, IRB No. 4–2020-1,071), and conducted in accordance with the Declaration of Helsinki. Given the retrospective design of the study and the use of anonymised patient data, the requirement for written informed consent was waived.

### 2.2. Data at AAV diagnosis

Data regarding AAV subtype, ANCA type and positivity, and AAV-specific indices were obtained from the medical records as AAV-specific data. AAV activity was assessed according to the Birmingham Vasculitis Activity Score (BVAS) and the prognosis was assessed using the five-factor score (FFS) (12, 13). Myeloperoxidase (MPO)-ANCA and proteinase 3 (PR3)-ANCA were measured by immunoassays, and perinuclear (P)-ANCA and cytoplasmic (C)-ANCA were assessed by an indirect immunofluorescence assay. They all were accepted as the method determining ANCA positivity or negativity (21, 22). Data regarding acute-phase reactants, erythrocyte sedimentation rate (ESR), and C-reactive protein (CRP) levels, were also collected. The remaining clinical and laboratory data are summarised in Table 1.

**Table 1:** Characteristics of AAV patients at diagnosis and during follow-up (*N* = 256).

Variables	Values
*At AAV diagnosis*	
Demographic data	
Age (years)	59.0 (20.8)
Male sex (N, (%))	90 (35.2)
BMI (kg/m^2^)	22.4 (4.2)
AAV subtype (*N*, (%))	
EGPA	52 (20.3)
GPA	69 (27.0)
MPA	135 (52.7)
ANCA type and positivity (*N*, (%))	
MPO-ANCA (or P-ANCA) positivity	170 (66.4)
PR3-ANCA (or C-ANCA) positivity	46 (18.0)
ANCA negativity	50 (19.5)
AAV-specific indices	
BVAS	12.0 (11.0)
FFS	1.0 (1.0)
Acute phase reactants	
ESR (mm/h)	59.0 (74.0)
CRP (mg/L)	14.2 (65.9)
Comorbidities (*N*, (%))	
DM	65 (25.4)
Hypertension	102 (39.8)
Dyslipidaemia	49 (19.1)
Laboratory results	
White blood cell count (/mm^3^)	9,180.0 (6,437.5)
Haemoglobin (g/dL)	11.4 (3.7)
Platelet count (× 10^9^/L)	299.5 (163.0)
Prothrombin time (INR)	1.0 (0.2)
Fasting glucose (mg/dL)	101.0 (35.3)
Blood urea nitrogen (mg/dL)	18.0 (18.8)
Serum creatinine (mg/dL)	0.9 (1.2)
Uric acid (mg/dL)	4.3 (2.5)
Total cholesterol (mg/dL)	168.0 (65.0)
Protein (g/dL)	6.7 (1.1)
Serum albumin (g/dL)	3.7 (1.1)
Liver-related variables	
ALP (IU/L)	73.0 (37.0)
AST (IU/L)	18.0 (9.0)
ALT (IU/L)	16.0 (14.0)
NFS	-4.7 (2.3)
*During the follow-up duration*	
All-cause mortality	
All-cause mortality (*N*, (%))	33 (12.9)
Follow-up duration based on all-cause mortality (months)	37.0 (65.4)
Medications (*N*, (%))	
Glucocorticoids	241 (94.1)
Cyclophosphamide	143 (55.9)
Rituximab	43 (16.8)
Mycophenolate mofetil	37 (14.5)
Azathioprine	137 (53.5)
Tacrolimus	22 (8.6)
Methotrexate	25 (9.8)

Values are expressed as a median (interquartile range, IQR) or *N* (%). AAV, ANCA-associated vasculitis; ANCA, antineutrophil cytoplasmic antibody; BMI, body mass index; EGPA, eosinophilic granulomatosis with polyangiitis; MPA, microscopic polyangiitis; GPA, granulomatosis with polyangiitis; MPO, myeloperoxidase; P, perinuclear; PR3, proteinase 3; C, cytoplasmic; BVAS, Birmingham vasculitis activity score; FFS, five-factor score; ESR, erythrocyte sedimentation rate; CRP, C-reactive protein; DM, diabetes mellitus; INR, international normalised ratio; ALP, alkaline phosphatase; AST, aspartate aminotransferase; ALT, alanine aminotransferase; NFS, non-alcoholic fatty liver disease fibrosis score.

### 2.3. NFS equation

NFS was calculated using the following equation (7, 8): NFS = −1.675 + 0.037 - age (years) + 0.094 - BMI (kg/m^2^) + 1.13 × impaired fasting glucose/DM (yes = 1, no = 0) + 0.99 × AST/ALT ratio - 0.013 × platelet count (×10^9^/L) - 0.66 × serum albumin (g/dl). The laboratory results which were measured or collected at the time of AAV diagnosis were used for the parameters of the equation for NFS. These values were collected before the treatment, and were not affected by medications.

### 2.4. All-cause mortality and medications administered during follow-up

All-cause mortality was defined as death due to any cause. Follow-up duration based on all-cause mortality was defined as the period between AAV diagnosis and the last visit for the surviving patients or as the period between AAV diagnosis and death among deceased patients (14, 17, 23). The frequency of glucocorticoids and immunosuppressive drug administration, including cyclophosphamide, rituximab, mycophenolate mofetil, azathioprine, tacrolimus, and methotrexate, was investigated during follow-up.

### 2.5. Statistical analysis

All statistical analyses were performed using SPSS version 26 (IBM Corporation, Armonk, NY, United States). Continuous variables are expressed as median with interquartile range, whereas categorical variables are expressed as number (percentage). Comparisons between categorical variables were performed using the chi-square and Fisher’s exact tests. The optimal cut-off value was extrapolated by performing receiver operator characteristic (ROC) curve analysis, and one value with the maximum sum of sensitivity and specificity was selected. The relative risk (RR) for the cut-off for high AAV activity was analysed using contingency tables and the chi-square test. The Mann–Whitney U test was used to compare continuous variables. A comparison of cumulative survival rates between groups was performed using Kaplan–Meier survival analysis with the log-rank test. Multivariable Cox hazards model analysis using variables with statistical significance in the univariable Cox hazard model was used to obtain hazard ratios (HRs) during follow-up. Differences with *p* < 0.05 were considered to be statistically significant.

## 3. Results

### 3.1. Characteristics

Regarding data at AAV diagnosis, the median age and BMI were 59.0 years and 22.4 kg/m2, respectively, and 35.2% of the patients were male. Of the 256 patients, 52, 69, and 135 patients were classified as having eosinophilic granulomatosis with polyangiitis, granulomatosis with polyangiitis, and microscopic polyangiitis, respectively. MPO-ANCA (or P-ANCA) and PR3-ANCA (or C-ANCA) were detected in 170 and 46 patients, respectively, whereas, ANCA was absent in 50 patients. The median BVAS, FFS, ESR and CRP levels were 12.0, 1.0, 59.0 mm/h, and 14.2 mg/L, respectively; the median NFS was-4.7. The remaining clinical and laboratory data are summarised in Table 1. Regarding data collected during follow-up of the 256 patients, 33 (12.9%) died during the corresponding median follow-up of 37.0 months. Glucocorticoids were administered to 241 patients. Among immunosuppressive drugs, cyclophosphamide (55.9%) was the most frequently administered drug, followed by azathioprine (53.5%) (Table 1). When DM, hypertension, and dyslipidaemia at AAV diagnosis and medications administered during follow-up were compared between surviving and deceased AAV patients, no statistically significant differences were observed between the two groups (Supplementary Table S1).

### 3.2. Optimal cut-off value of NFS for all-cause mortality and relative risk

**Figure 1:**
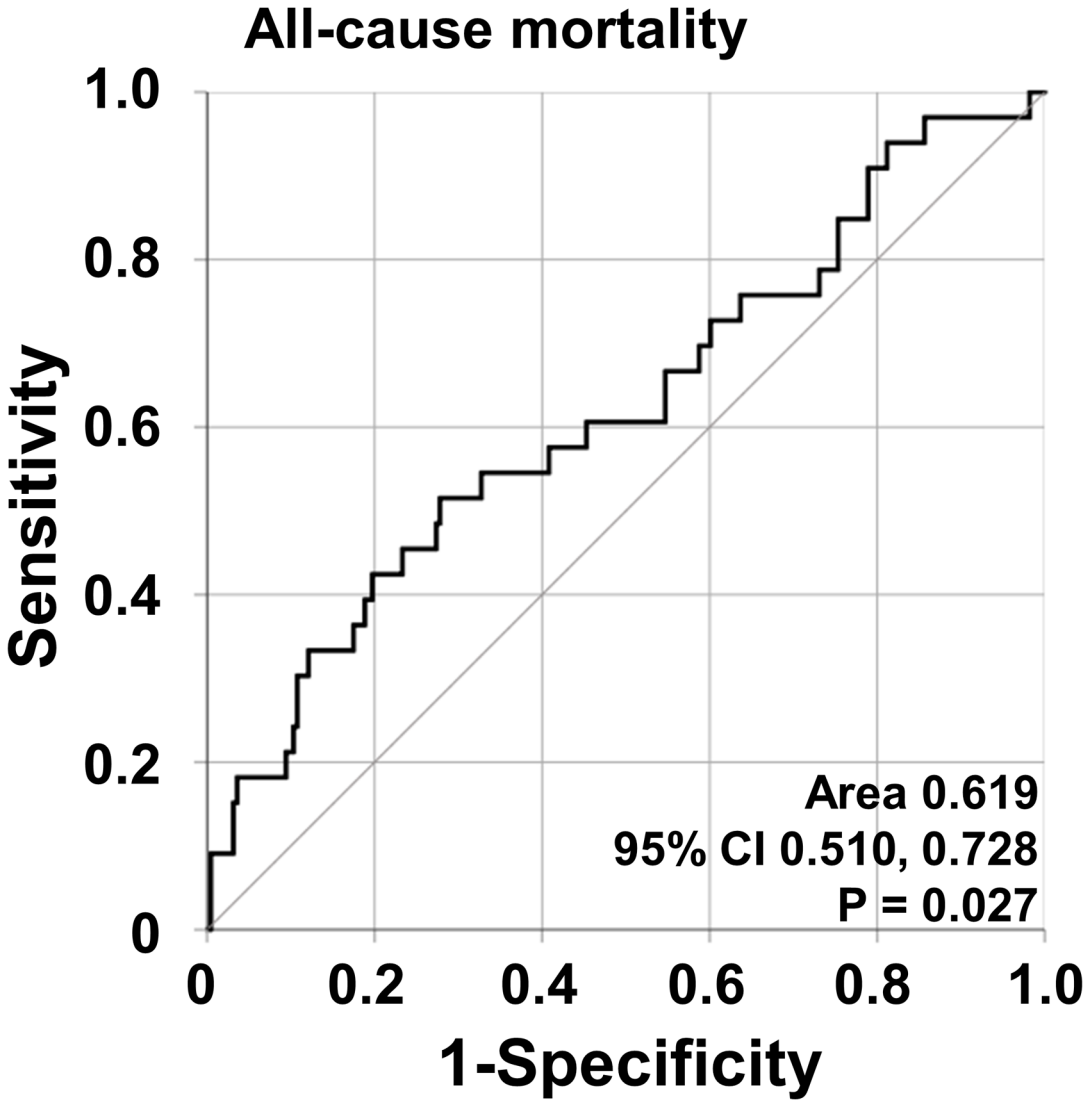
Optimal cut-off values for NFS and relative risks. The ROC curve for calculating an optimal cut-off of NFS for all-cause mortality. NFS, non-alcoholic fatty liver disease fibrosis score; ROC, receiver operating characteristic.

According to the ROC curve analysis, the optimal cut-off of NFS for all-cause mortality was-3.97 (sensitivity 51.5% and specificity 72.2%) (area under the ROC curve [AUC] 0.619, 95% confidence interval [CI] 0.510, 0.728) (Figure 1). All-cause mortality was significantly higher for patients with NFS ≥ −3.97 compared to that of below (21.5% vs. 9.0%, respectively) and patients with NFS ≥ −3.97 exhibited a significantly increased risk of all-cause mortality compared to those with NFS < −3.97 (RR 2.759, 95% CI 1.313, 5.800) (Supplementary Figure S1).

### 3.3. Comparison of cumulative patients’ survival rates

**Figure 2:**
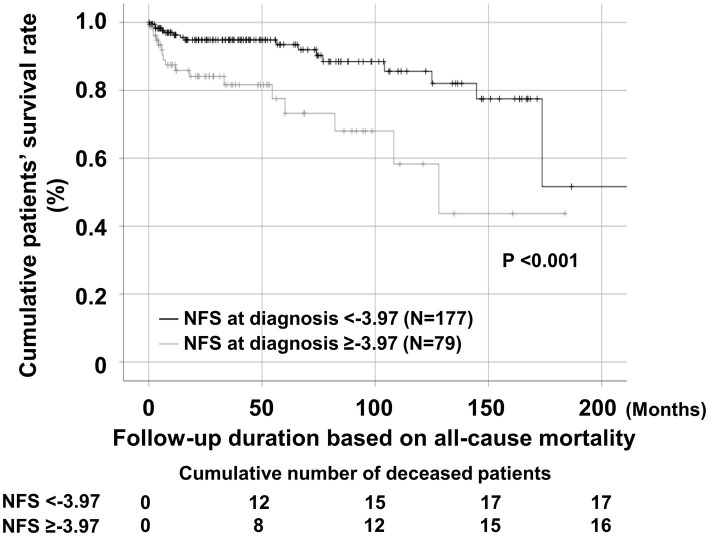
Comparison of cumulative patients’ survival rates. Comparison of cumulative patient survival rates between AAV patients with NFS at diagnosis ≥ − 3.97 and those with NFS at diagnosis <−3.97. AAV, antineutrophil cytoplasmic antibody-associated vasculitis; NFS, non-alcoholic fatty liver disease fibrosis score.

Regarding all-cause mortality, AAV patients with NFS at diagnosis ≥ − 3.97 exhibited a significantly lower cumulative patients’ survival rate than those with NFS at diagnosis <−3.97 (*p* < 0.001) (Figure 2).

### 3.4. Cox analyses for all-cause mortality

The univariable Cox analysis revealed that age, male sex, BVAS, FFS, ESR, CRP, haemoglobin, prothrombin time, blood urea nitrogen, serum creatinine, uric acid, total cholesterol, protein, serum albumin, alkaline phosphatase, AST, NFS, and NFS ≥ −3.97 at diagnosis were significantly associated with all-cause mortality during follow-up of the patients. The multivariable Cox analysis using NFS at diagnosis revealed that age (HR 1.039), male sex (HR 2.811), BVAS (HR 1.100), FFS (HR 1.607), uric acid (HR 1.366), and serum albumin (HR 0.332) were significantly and independently associated with all-cause mortality during follow-up. However, NFS at diagnosis itself was not independently associated with all-cause mortality. Meanwhile, the multivariable Cox analysis using NFS at diagnosis ≥ − 3.97 revealed that male sex (HR 2.714), BVAS (HR 1.112), FFS (HR 1.737), uric acid (HR 1.354), and serum albumin (HR 0.335) were significantly and independently associated with all-cause mortality during follow-up. In particular, NFS at diagnosis ≥ − 3.97 (HR 2.232, 95% CI 1.011, 4.925) was independently associated with all-cause mortality among AAV patients (Table 2).

**Table 2:** Cox hazards model analyses of NFS and other variables at diagnosis for all-cause mortality during follow-up in AAV patients.

Variables	Univariable			Multivariable (NFS)			Multivariable (NFS ≥ −3.97)		
	HR	95% CI	*p* value	HR	95% CI	*p* value	HR	95% CI	*p* value
Age (years)	1.065	1.031, 1.100	<0.001	1.039	1.002, 1.077	0.039	1.036	0.999, 1.074	0.054
Male sex (N, (%))	2.404	1.207, 4.788	0.013	2.811	1.199, 6.589	0.017	2.714	1.161, 6.346	0.021
BMI (kg/m^2^)	1.112	0.995, 1.241	0.061						
MPO-ANCA (or P-ANCA) positivity	1.737	0.801, 3.766	0.162						
PR3-ANCA (or C-ANCA) positivity	0.764	0.294, 1.987	0.581						
BVAS	1.104	1.055, 1.156	<0.001	1.100	1.030, 1.176	0.005	1.112	1.039, 1.190	0.002
FFS	2.122	1.518, 2.967	<0.001	1.607	1.033, 2.500	0.036	1.737	1.099, 2.744	0.018
ESR (mm/h)	1.009	1.000, 1.018	0.039	0.992	0.978, 1.007	0.294	0.992	0.978, 1.006	0.266
CRP (mg/L)	1.008	1.003, 1.013	0.002	0.999	0.991, 1.008	0.891	0.999	0.990, 1.008	0.825
DM	1.013	0470, 2.183	0.974						
Hypertension	1.015	0.509, 2.026	0.966						
Dyslipidaemia	1.721	0.796, 3.721	0.168						
White blood cell count (/mm^3^)	1.000	1.000, 1.000	0.055						
Haemoglobin (g/dL)	0.776	0.661, 0.911	0.002	1.010	0.780, 1.308	0.939	1.036	0.802, 1.338	0.785
Platelet count (× 10^9^/L)	1.000	0.998, 1.002	0.945						
Prothrombin time (INR)	20.495	1.183, 355.071	0.038	0.355	0.005, 23.485	0.628	0.489	0.007, 32.151	0.737
Fasting glucose (mg/dL)	1.005	0.998, 1.012	0.159						
Blood urea nitrogen (mg/dL)	1.011	1.002, 1.019	0.012	0.982	0.962, 1.003	0.096	0.985	0.964, 1.006	0.149
Serum creatinine (mg/dL)	1.140	1.004, 1.295	0.043	0.924	0.706, 1.209	0.563	0.913	0.702, 1.188	0.500
Uric acid (mg/dL)	1.220	1.059, 1.405	0.006	1.366	1.089, 1.713	0.007	1.354	1.082, 1.694	0.008
Total cholesterol (mg/dL)	0.990	0.981, 0.998	0.017	0.999	0.989, 1.010	0.885	1.000	0.989, 1.010	0.941
Protein (g/dL)	0.588	0.386, 0.897	0.014	0.977	0.863, 1.107	0.718	0.973	0.860, 1.101	0.663
Serum albumin (g/dL)	0.399	0.205, 0.560	<0.001	0.332	0.149, 0.736	0.007	0.335	0.153, 0.734	0.006
ALP (IU/L)	1.003	1.001, 1.006	0.010	1.004	0.998, 1.010	0.153	1.005	0.999, 1.010	0.110
AST (IU/L)	1.012	1.002, 1.022	0.023	0.996	0.981, 1.011	0.579	0.994	0.978, 1.009	0.417
ALT (IU/L)	1.004	0.996, 1.012	0.347						
NFS	1.334	1.096, 1.623	0.004	1.164	0.943, 1.437	0.158			
NFS ≥ -3.97	3.214	1.610, 6.416	0.001				2.232	1.011, 4.925	0.047

NFS, non-alcoholic fatty liver disease fibrosis score; AAV, ANCA-associated vasculitis; ANCA, antineutrophil cytoplasmic antibody; HR: hazard ratio; CI, confidence interval; BMI, body mass index; MPO, myeloperoxidase; P, perinuclear; PR3, proteinase 3; C, cytoplasmic; BVAS, Birmingham vasculitis activity score; FFS, five-factor score; ESR, erythrocyte sedimentation rate; CRP, C-reactive protein; DM, diabetes mellitus; INR, international normalised ratio; ALP, alkaline phosphatase: AST, aspartate aminotransferase; ALT, alanine aminotransferase.

## 4. Discussion

The results of the present longitudinal cohort study revealed that patients with high NFS were at higher risk for all-cause mortality than those with low NFS at AAV diagnosis, even after adjusting for various confounding factors. We found a positive relationship between NFS at AAV diagnosis and all-cause mortality during follow-up of AAV patients. This finding suggests that even non-significant liver fibrosis assessed according to NFS may indicate an increased risk for mortality among patients with vasculitis. This also suggests that NFS can be used as a useful predictor of poor outcomes in AAV patients. To our knowledge, this is the first study to demonstrate that NFS at AAV diagnosis could predict all-cause mortality during follow-up in AAV patients.

By what mechanism could NFS predict all-cause mortality? Recent studies have explained possible mechanisms underlying the association between liver fibrosis and mortality. Individuals with liver fibrosis exhibit higher plasma levels of inflammatory and haemostatic factors, hyperuricaemia, lower circulating insulin-like growth factor-1 levels, endothelial dysfunction, and biomarkers of oxidative stress that lead to poor outcomes such as cardiovascular disease (24–27). However, in this study, even patients who appeared to not have significant liver fibrosis (−3.97 ≤ NFS < −1.455) were at a higher risk for mortality. Therefore, other explanations are needed to clarify the positive association between NFS and the risk for mortality. The equation for NFS comprises six parameters with positive coefficients assigned to four (age, BMI, DM, and AST/ALT ratio), and negative coefficients assigned to two (platelet count and serum albumin).

Of the four parameters assigned positive coefficients, age, and DM are well-known traditional risk factors for all-cause mortality (28). In this study, age appeared to increase the rate of all-cause mortality in the univariable Cox analysis; however, the multivariable analysis failed to reveal statistical significance. DM was not associated with all-cause mortality among AAV patients (Table 2). On the other hand, the association between BMI and all-cause mortality is known to exhibit a J-shaped pattern: obese (BMI >30 kg/m2) and underweighted (BMI <18.5 kg/m2) individuals exhibit significantly higher mortality rates than healthy-weighted individuals (BMI 18.5–24.9 Kg/m2) (29). This pattern may explain one result of this study in that BMI almost tended to be associated with all-cause mortality, although it was not statistically significant (Table 2).

The AST/ALT ratio provides an important clue to a diagnostic approach to liver dysfunction, there have also been reports addressing the association between the AST/ALT ratio and all-cause mortality among individuals without chronic liver diseases (30, 31). In the present study, the univariable Cox analysis revealed that the AST/ALT ratio similarly demonstrated the potential to predict all-cause mortality (HR 1.679, 95% CI 1.133, 2.489). In addition, of the two parameters assigned negative coefficients, serum albumin has been reported to be a predictor of all-cause mortality among elderly individuals (32), and was demonstrated to independently predict all-cause mortality among AAV patients in the present study. However, platelet count was not significantly associated with all-cause mortality in this study (Table 2). In summary, of the six parameters, serum albumin and the AST/ALT ratio demonstrated a significant (*p* < 0.05) association with all-cause mortality, while age and BMI also demonstrated a fairly notable contribution. As such, NFS may have a clear theoretical basis for predicting all-cause mortality among AAV patients in addition to NAFLD and liver fibrosis.

According to the previous studies, individuals with NFS < -1.455 could not be considered to have significantly advanced liver fibrosis. Given that AAV rarely involves the liver and advanced liver fibrosis could affect the results of this study, we excluded seven patients with NFS ≥ -1.455, and re-examined the clinical implications of NFS in 249 AAV patients without significantly advanced liver fibrosis or indeterminate fibrosis score (7, 8). According to the ROC curve analysis, the optimal cut-off value for NFS for all-cause mortality was-3.97 (sensitivity 46.7% and specificity 73.5%), which is the same cut-off value shown in Supplementary Figure S1. When AAV patients were divided into two groups according to NFS of −3.97, all-cause mortality was found in those with NFS at diagnosis ≥ − 3.97 more commonly than those with NFS at diagnosis <−3.97 (19.4% vs. 9.0%). AAV patients with NFS at diagnosis ≥ − 3.97 exhibited a significantly higher risk for all-cause mortality than those with NFS at diagnosis <−3.97 (RR 2.429, 95% CI 1.116, 5.286) (Supplementary Figure S2). When the cumulative survival rates were compared, AAV patients with NFS at diagnosis ≥ − 3.97 exhibited a significantly lower patients’ survival rate than those with NFS at diagnosis <−3.97 (*p* = 0.004) (Supplementary Figure S3).

In addition, the multivariable Cox analysis including variables that were statistically significant in the univariable analysis revealed that male sex (HR 2.495), BVAS (HR 1.136), FFS (HR 1.819), uric acid (HR 1.331), and serum albumin (HR 0.378) were significantly and independently associated with all-cause mortality during follow-up. Furthermore, NFS at diagnosis ≥ − 3.97 (HR 2.934, 95% CI 1.220, 7.053) was also independently associated with all-cause mortality in AAV patients (Supplementary Table S2). Therefore, it is concluded that NFS at AAV diagnosis can be applied to AAV patients to predict all-cause mortality regardless of the degree of liver fibrosis.

The primary strength of this study is that we investigated whether NFS at AAV diagnosis could predict poor prognosis of AAV and demonstrated that it could predict all-cause mortality during follow-up in AAV patients for the first time.

### 4.1. Limitations

The present study had several limitations. First, although we used data from a prospective cohort of AAV patients, it was conducted retrospectively by reviewing medical records. In addition, its single-centre design was another limitation, despite low inter-observer variations. For this reason, gamma-glutamyl transferase, a variable reflecting liver function, could not be evaluated because of missing data, and the fact that the number of patients was small could not be ignored. Also, since the study population was limited to Korean, the data could only apply to East Asian population at the best. We could not incorporate inter-racial characteristics in the analysis. Nevertheless, given the strengths of this study, our results have clinical significance similar to a pilot study. Future prospective studies with a larger number of patients, as well as those with serial assessments of NFS and liver-related variables, will clarify and validate the clinical implications of NFS for predicting all-cause mortality among AAV patients.

## 5. Conclusion

In conclusion, this study is the first to demonstrate that NFS at AAV diagnosis was clinically useful in predicting all-cause mortality during follow-up in AAV patients without substantial chronic liver diseases. Therefore, we expect that NFS calculated at AAV diagnosis will be an additional index for poor outcomes of AAV in addition to estimating the extent of NAFLD in AAV patients.

## Data availability statement

The raw data supporting the conclusions of this article will be made available by the authors, without undue reservation.

## Ethics statement

The studies involving humans were approved by Institutional Review Board (IRB) of Severance Hospital (Seoul, Republic of Korea, IRB No. 4-2020-1071). The studies were conducted in accordance with the local legislation and institutional requirements. The ethics committee/institutional review board waived the requirement of written informed consent for participation from the participants or the participants' legal guardians/next of kin because given the retrospective design of the study and the use of anonymised patient data, the requirement for written informed consent was waived.

## Author contributions

JW and PP carried out the statistical analysis. JW, PP and S-WL wrote the first draft of the manuscript. JW, PP, Y-BP, JH, and S-WL collected data, corrected, and approved the revisions and final version of the manuscript. JH and S-WL are responsible for the conception, funding, and design of the study and guarantors of this work and, as such, had full access to all the data in the study and take responsibility for the integrity of the data and the accuracy of the data analysis. All authors contributed to the article and approved the submitted version.

## Funding

This study received funding from the Korea Health Technology R&D Project through the Korea Health Industry Development Institute, funded by the Ministry of Health and Welfare (HI14C1324), Handok Inc., Seoul, Republic of Korea (HANDOK 2021–006), and CELLTRION PHARM, Inc. Chungcheongbuk-do, Republic of Korea (NCR 2019–6). The funders were not involved in the study design, collection, analysis, interpretation of data, the writing of this article or the decision to submit it for publication.

## Conflict of interest

The authors declare that the research was conducted in the absence of any commercial or financial relationships that could be construed as a potential conflict of interest.

## Publisher’s note

All claims expressed in this article are solely those of the authors and do not necessarily represent those of their affiliated organizations, or those of the publisher, the editors and the reviewers. Any product that may be evaluated in this article, or claim that may be made by its manufacturer, is not guaranteed or endorsed by the publisher.
