# Supplementary material for: Non-alcoholic fatty liver disease fibrosis score is a useful index for predicting all-cause mortality in patients with antineutrophil cytoplasmic antibody-associated vasculitis
Source: Front Med (Lausanne). 2023 Aug 29;10:1217937. doi: 10.3389/fmed.2023.1217937 (PMC10497776; doi:10.3389/fmed.2023.1217937)
Supplement: Supplementary file 1 [file Data_Sheet_1.docx]

Supplementary Material

# Supplementary Data

Supplementary Material should be uploaded separately on submission. Please include any supplementary data, figures and/or tables. All supplementary files are deposited to FigShare for permanent storage and receive a DOI.

Supplementary material is not typeset so please ensure that all information is clearly presented, the appropriate caption is included in the file and not in the manuscript, and that the style conforms to the rest of the article. To avoid discrepancies between the published article and the supplementary material, please do not add the title, author list, affiliations or correspondence in the supplementary files.

# Supplementary Figures and Tables

For more information on Supplementary Material and for details on the different file types accepted, please see [here](http://home.frontiersin.org/about/author-guidelines#SupplementaryMaterial). Figures, tables, and images will be published under a Creative Commons CC-BY licence and permission must be obtained for use of copyrighted material from other sources (including re-published/adapted/modified/partial figures and images from the internet). It is the responsibility of the authors to acquire the licenses, to follow any citation instructions requested by third-party rights holders, and cover any supplementary charges.

## Supplementary Figures


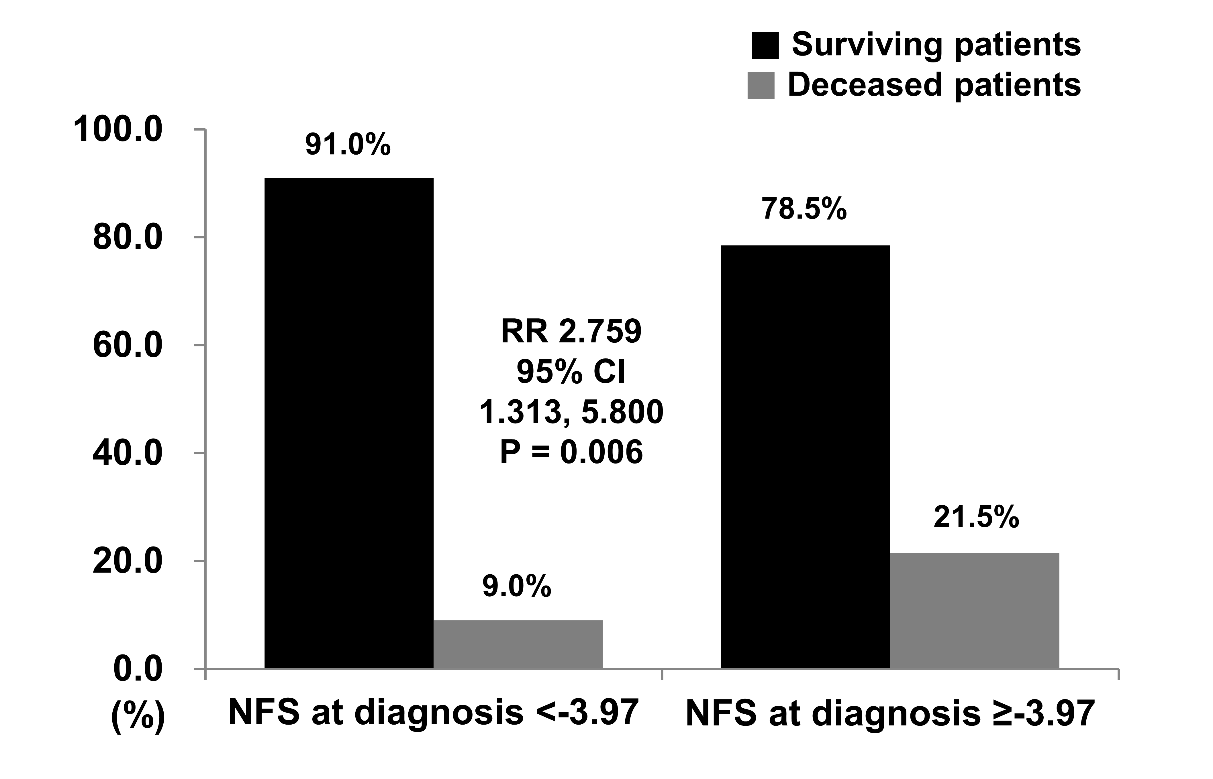


**Supplementary Figure 1.** Optimal cut-off values for NFS and relative risks in 249 AAV patients. Using the ROC curve, the optimal cut-off value for NFS at diagnosis for all-cause mortality was -3.97. When AAV patients were divided into two groups according to NFS of -3.97, all-cause mortality was found in those with NFS at diagnosis ≥-3.97 more commonly than those with NFS at diagnosis <-3.97. AAV patients with NFS at diagnosis ≥-3.97 exhibited a significantly higher risk for all-cause mortality than those with NFS at diagnosis <-3.97 (RR 2.759). NFS: non-alcoholic fatty liver disease fibrosis score; AAV: antineutrophil cytoplasmic antibody-associated vasculitis; ROC: receiver operating characteristic; RR: relative risk.


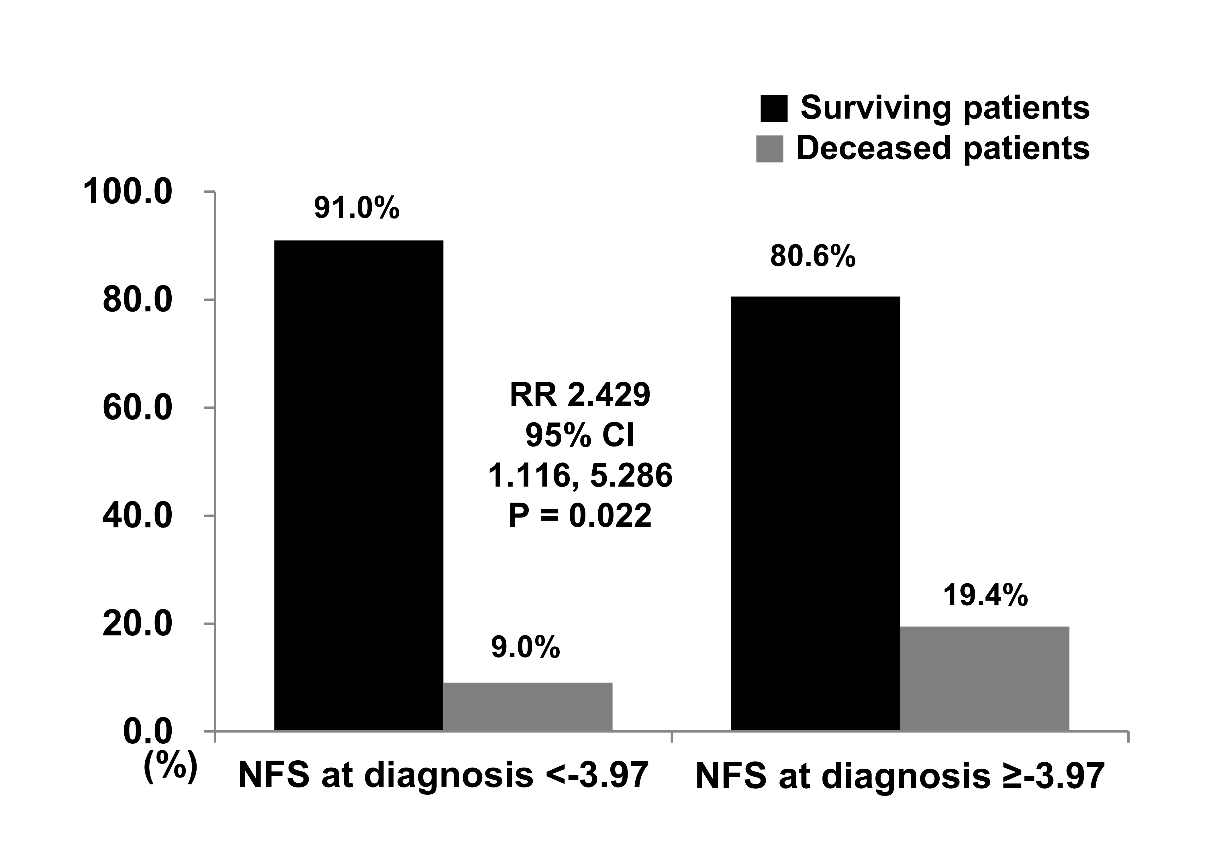


**Supplementary Figure 2.** Optimal cut-off values for NFS and relative risks in 249 AAV patients without significantly advanced liver fibrosis. Using the ROC curve, the optimal cut-off value for NFS at diagnosis for all-cause mortality was -3.97. When AAV patients were divided into two groups according to NFS of -3.97, all-cause mortality was found in those with NFS at diagnosis ≥-3.97 more commonly than those with NFS at diagnosis <-3.97. AAV patients with NFS at diagnosis ≥-3.97 exhibited a significantly higher risk for all-cause mortality than those with NFS at diagnosis <-3.97 (RR 2.429). NFS: non-alcoholic fatty liver disease fibrosis score; AAV: antineutrophil cytoplasmic antibody-associated vasculitis; ROC: receiver operating characteristic; RR: relative risk.


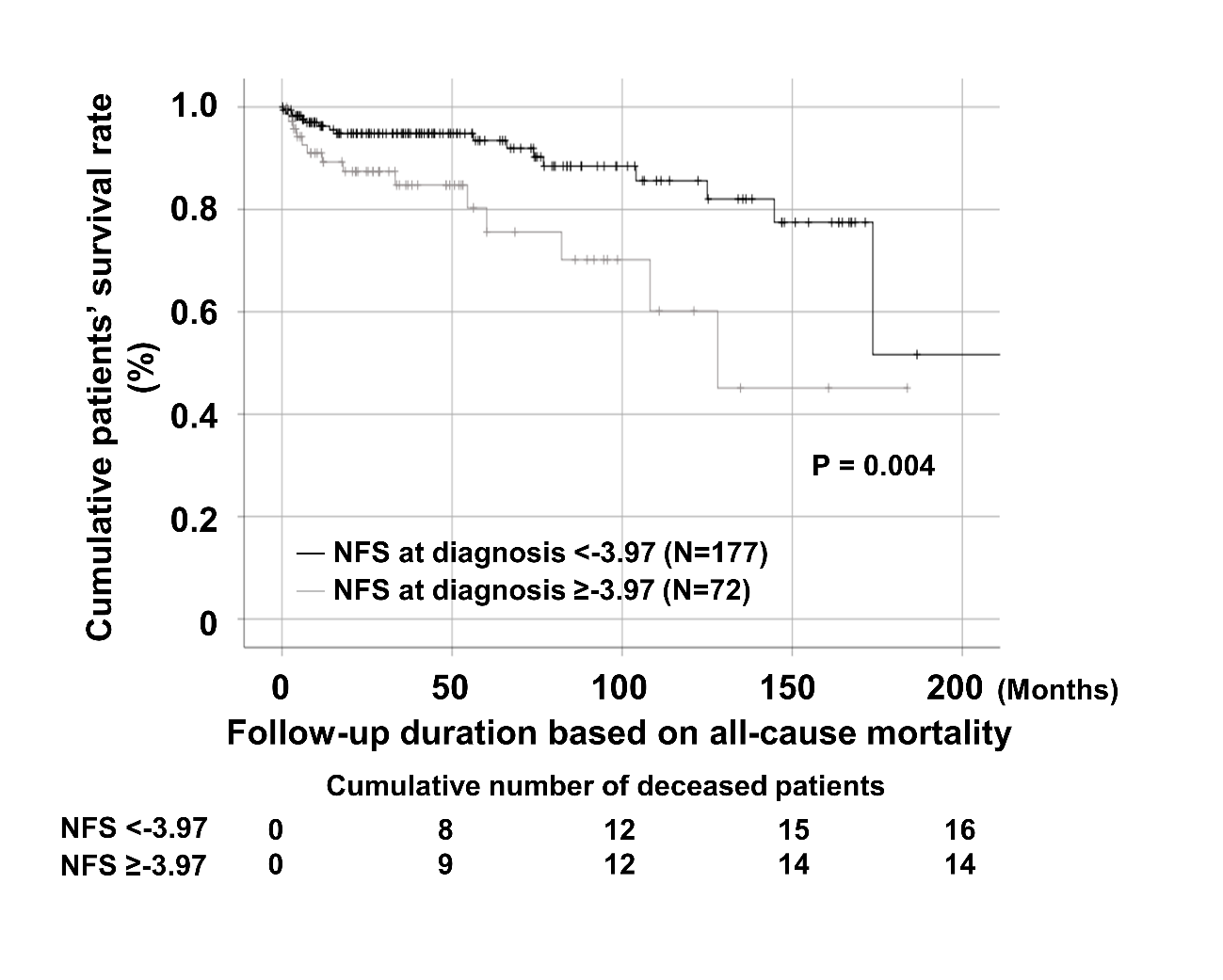


**Supplementary Figure 3**. Comparison of cumulative patients’ survival rates. When the cumulative survival rates were compared, AAV patients with NFS at diagnosis ≥-3.97 exhibited a significantly lower patients’ survival rate than those with NFS at diagnosis <-3.97 (P = 0.004). AAV: antineutrophil cytoplasmic antibody-associated vasculitis; NFS: non-alcoholic fatty liver disease fibrosis score.

## Supplementary Tables

**Supplementary Table 1. Comparison of comorbidities at AAV diagnosis and medications administered during follow-up between surviving AAV patients and deceased ones**

| **Variables** | **Surviving patients**  **(N=223)** | **Deceased patients**  **(N=33)** | **P-value** |
| --- | --- | --- | --- |
| ***At diagnosis*** |  |  |  |
| **Comorbidities (N, (%))** |  |  |  |
| DM | 56 (25.1) | 9 (27.3) | 0.790 |
| Hypertension | 88 (39.5) | 14 (42.4) | 0.746 |
| Dyslipidaemia | 40 (17.9) | 9 (27.3) | 0.203 |
| ***During the follow-up duration*** |  |  |  |
| **Medications (N, (%))** |  |  |  |
| Glucocorticoids | 208 (93.3) | 33 (100) | 0.125 |
| Cyclophosphamide | 124 (55.6) | 19 (57.6) | 0.832 |
| Rituximab | 35 (15.7) | 8 (24.2) | 0.220 |
| Mycophenolate mofetil | 33 (14.8) | 4 (12.1) | 0.683 |
| Azathioprine | 122 (54.7) | 15 (45.5) | 0.320 |
| Tacrolimus | 20 (9.0) | 2 (6.1) | 0.578 |
| Methotrexate | 24 (10.8) | 1 (3.0) | 0.163 |

Values are expressed as a median (interquartile range, IQR) or N (%).

ANCA: antineutrophil cytoplasmic antibody; AAV: ANCA-associated vasculitis; DM: diabetes mellitus.

**Supplementary Table 2 Cox hazards model analyses of NFS and other variables at AAV diagnosis for all-cause mortality in 249 AAV patients without significantly advanced liver fibrosis**

| **Variables** | **Univariable** | | |  | **Multivariable**  **(NAFLD fibrosis score ≥ -3.97)** | | |
| --- | --- | --- | --- | --- | --- | --- | --- |
|  | **HR** | **95% CI** | **P value** |  | **HR** | **95% CI** | **P value** |
| Age (years) | 1.060 | 1.026, 1.096 | 0.001 |  | 1.002 | 0.985, 1.061 | 0.243 |
| Male sex (N, (%)) | 2.303 | 1.118, 4.744 | 0.024 |  | 2.495 | 1.048, 5.940 | 0.039 |
| BMI (kg/m^2^) | 1.156 | 1.030, 1.297 | 0.014 |  | 1.115 | 0.971, 1.280 | 0.123 |
| MPO-ANCA (or P-ANCA) positivity | 1.568 | 0.712, 3.454 | 0.264 |  |  |  |  |
| PR3-ANCA (or C-ANCA) positivity | 0.839 | 0.319, 2.201 | 0.721 |  |  |  |  |
| BVAS | 1.101 | 1.049, 1.155 | <0.001 |  | 1.136 | 1.051, 1.228 | 0.001 |
| FFS | 2.129 | 1.500, 3.021 | <0.001 |  | 1.819 | 1.132, 2.924 | 0.013 |
| ESR (mm/hr) | 1.011 | 1.002, 1.020 | 0.020 |  | 0.999 | 0.985, 1.014 | 0.931 |
| CRP (mg/L) | 1.008 | 1.002, 1.013 | 0.004 |  | 0.997 | 0.989, 1.006 | 0.576 |
| DM | 0.976 | 0.434, 2.197 | 0.953 |  |  |  |  |
| Hypertension | 0.926 | 0.446, 1.925 | 0.838 |  |  |  |  |
| Dyslipidaemia | 1.419 | 0.606. 3.324 | 0.420 |  |  |  |  |
| White blood cell count (/mm^3^) | 1.000 | 1.000, 1.000 | 0.061 |  |  |  |  |
| Haemoglobin (g/dL) | 0.807 | 0.683, 0.953 | 0.012 |  | 1.095 | 0.848, 1.414 | 0.488 |
| Platelet count (× 10^9^/L) | 1.000 | 0.998, 1.003 | 0.705 |  |  |  |  |
| Prothrombin time (INR) | 21.433 | 1.067, 430.409 | 0.045 |  | 2.237 | 0.054, 92.004 | 0.671 |
| Fasting glucose (mg/dL) | 1.004 | 0.997, 1.012 | 0.257 |  |  |  |  |
| Blood urea nitrogen (mg/dL) | 1.009 | 1.000, 1.018 | 0.048 |  | 0.980 | 0.960, 1.000 | 0.054 |
| Serum creatinine (mg/dL) | 1.116 | 0.973, 1.281 | 0.117 |  |  |  |  |
| Uric acid (mg/dL) | 1.225 | 1.058, 1.420 | 0.007 |  | 1.331 | 1.081, 1.638 | 0.007 |
| Total cholesterol (mg/dL) | 0.990 | 0.982, 0.999 | 0.034 |  | 1.000 | 0.990, 1.010 | 0.979 |
| Protein (g/dL) | 0.662 | 0.428, 1.025 | 0.064 |  |  |  |  |
| Serum albumin (g/dL) | 0.355 | 0.211, 0.597 | <0.001 |  | 0.378 | 0.172, 0.831 | 0.016 |
| ALP (IU/L) | 1.002 | 0.999, 1.006 | 0.128 |  |  |  |  |
| AST (IU/L) | 1.011 | 0.999, 1.022 | 0.064 |  |  |  |  |
| ALT (IU/L) | 1.004 | 0.997, 1.012 | 0.259 |  |  |  |  |
| NFS ≥ -3.97 | 2.796 | 1.354, 5.775 | 0.005 |  | 2.934 | 1.220, 7.053 | 0.016 |

NFS: non-alcoholic fatty liver disease fibrosis score; AAV: ANCA-associated vasculitis; ANCA: antineutrophil cytoplasmic antibody; HR: hazard ratio; CI: confidence interval; BMI: body mass index; MPO: myeloperoxidase; P: perinuclear; PR3: proteinase 3; C: cytoplasmic; BVAS: Birmingham vasculitis activity score; FFS: five-factor score; ESR: erythrocyte sedimentation rate; CRP: C-reactive protein; DM: diabetes mellitus; INR: international normalised ratio; ALP: alkaline phosphatase: AST: aspartate aminotransferase; ALT: alanine aminotransferase.
